# Association between magnesium concentrations and prediabetes: a systematic review and meta-analysis

**DOI:** 10.1038/s41598-021-03915-3

**Published:** 2021-12-22

**Authors:** Sara Ebrahimi Mousavi, Seyed Mojtaba Ghoreishy, Amirhossein Hemmati, Hamed Mohammadi

**Affiliations:** Department of Clinical Nutrition, School of Nutritional Sciences and Dietetics, Tehran University of Medical Sciences, Tehran, Iran

**Keywords:** Diseases, Metabolic disorders

## Abstract

Studies on the association between serum magnesium level and prediabetes yielded inconsistent results. Therefore, the present meta-analysis was designed to examine the association between serum magnesium levels and prediabetes. Online databases including PubMed, Embase, Scopus and Google Scholar were searched up to October, 2020. A total of 10 studies that reported mean and standard deviation (SD) of magnesium levels in prediabetes and healthy control group were identified. Random effects models were used to pool weighted mean differences (WMDs) of serum magnesium levels. Pooled-analysis showed that subjects with prediabetes had significantly lower serum magnesium levels compared with healthy controls (WMD =  − 0.07 mmol/L; 95% CI − 0.09, − 0.05 mmol/L, P < 0.001). A significant heterogeneity observed across included studies (I^2^ = 95.6%, P < 0.001). However, different subgroup analysis did not detect the potential source of observed heterogeneity. Withdrawal of each individual study had no effect on the overall results. The present meta-analysis showed that circulating magnesium levels in people with prediabetes were significantly lower than healthy controls, confirming that magnesium deficiency may play a role in development and progression of prediabetes. Further studies with larger sample size and robust design are warranted to confirm present results.

## Introduction

Prediabetes is defined as an intermediate state of hyperglycemia with glycemic parameters between normal and diabetes threshold^1^. To date, no laboratory threshold has been agreed for the diagnosis of prediabetes. According to the American Diabetic Association (ADA), prediabetes is defined as impaired fasting glucose (IFG) of 5.6–6.9 mmol/L and/or 2 h post-challenge glucose of 7.8–11.0 mmol/L with a 75 g oral glucose tolerance test (impaired glucose tolerance [IGT]) or based on a HbA1c value of 5.7–6.4%^2^. However, the World Health Organization (WHO) has set a prediabetes threshold of IFG 6.1–6.9 mmol/L^3^. The lower IFG threshold in the ADA criterion is due to the increased risks of micro- and macro-vascular complications near this threshold^4^. ADA criteria overestimates the prevalence of prediabetes and include more people who are at higher risk for diabetes and cardiovascular disease^5,6^. It has been suggested that individuals with glucose metabolic disorders had altered mineral metabolism^7^. In this regard, magnesium has a crucial role in development and progression of chronic disorders such as type 2 diabetes and cardiovascular diseases^8–10^. Magnesium is an important cofactor in many biochemical reactions and also plays a role in regulating a number of vital functions such as muscle contraction, neuromuscular conduction, glucose control, myocardial electrical activity and blood pressure^11–14^. Recently, two meta-analysis have shown a beneficial role of magnesium supplementation on glucose parameters and insulin sensitivity in people with or at risk of diabetes^15–17^.

Previous studies that investigated the associations between the circulating levels of magnesium and prediabetes have yielded inconsistent results. Some studies documented the lower levels of magnesium in people with prediabetes compared to their healthy controls^18–20^, while others did not find significant differences^21,22^. Till now, there is no meta-analyses to address these inconsistent results. Therefore, the present systematic review and meta-analysis was designed to quantitatively examine the association between circulating magnesium levels and prediabetes.

## Methods

This study was performed based on the PRISMA (Preferred Reporting Items for Systematic Reviews and Meta-Analyses) protocol for reporting systematic reviews and meta-analyses^23^.

### Search strategy

Online databases including Medline, Embase, Scopus and Google scholar were searched up to October 2020, without limitation in publication time and language. The following key words were used in the current study: “Magnesium” AND "Prediabetic state” OR "Impaired glucose tolerance” OR "prediabetes" OR "Prediabetes” OR “Hyperglycaemia" OR” Borderline diabetes". In addition, we manually checked all reference lists of included articles and related reviews^24,25^ to avoid missing any relevant studies (Supplementary Table 1).

### Inclusion and exclusion criteria

All original observational case–control and cross-sectional studies were included if they: (1) examined the levels of magnesium in prediabetes individuals; and (2) provided sufficient data on serum/plasma magnesium levels in both prediabetes and control groups. papers were excluded if they: (1) enrolled patients with a disease other than prediabetes (2) without healthy control group, (3) published in non-English language or reported duplicate data; and (4) were reviews, conference abstract, clinical trials, letters, editorial articles, or case reports.

### Data extraction

Two independent investigators (SEB and SMG) extracted the relevant data and third investigator (HM) resolve any disagreements. The following information were extracted: author, publication year, country, number of cases and controls, mean age, criteria of prediabetes, magnesium levels (mean ± SD), magnesium assessment method, and study design.

### Quality of assessment

To evaluate the quality of the case–control studies, we used the Newcastle–Ottawa Scale (NOS) to evaluate the following characteristics with a maximum of nine starts for each study^26^.

A. Selection (4 items): adequacy of case definition; representativeness of the cases; selection of controls; and definition of controls.
B. Comparability (1 item): comparability of cases and controls on the basis of the design or analysis. According to the confounders including age, sex, the comparability of included studies was assessed. If a study adjusted its results for age, one star was given to it. Also, two stars were given to a study which adjusted its findings for age and sex.
C. Exposure (3 items): ascertainment of exposure; same method of ascertainment for cases and controls; and non-response rate (same rate for both groups).

Also, the evaluation of the quality of cross-sectional studies with the modified version of NOS was performed by evaluating the following characteristics with a maximum of ten starts for each study^27^.

A. Selection (4 items): representativeness of samples; sample size; non-respondents characteristics; ascertainment of the exposure. To assess exposure, if a study has used the validated method, it receives two stars and if it is not validated, it receives one star.
B. Comparability (1 item): comparability of two groups on the basis of the design or analysis. According to the confounders including age, sex, the comparability of included studies was assessed. As in the case–control studies, it received one star if the study results were controlled for age only, and two stars if adjusted for age and sex.
C. Outcome (3 items): assessment of the outcome: statistical test. To evaluate the result, if a study was evaluated using a record linkage or independent blind evaluation, it received two stars and, in the absence of a description, one star.

Articles with a total score of 0–4, 5–7, and 8–10 were considered as low, moderate and high quality, respectively.

### Statistical analysis

Statistical analysis was performed by using Stata 14.0 and P ≤ 0.05 was considered statistically significant. The overall relationship between serum and plasma magnesium levels and prediabetes was calculated by comparing the mean and standard deviation (SD) of magnesium levels in prediabetes compared with the healthy control group. When a study provided standard error (SE), SD was calculated by using this equation: (SD = SE × square root [number of participants]). Also, if a study reported medians and ranges or 95% CIs, we computed mean (SD) by Hozo’s method^28^. Prior the analysis of effect size, the levels of magnesium in serum and plasma were converted to mmol/L. Weighted mean differences (WMDs) with 95% CIs in magnesium levels were estimated by a random-effects model^29^. Heterogeneity between included studies was assessed by Cochran's Q test and the I^2^ statistic^30^. Subgroup analysis was performed to find a possible source of heterogeneity. In order to evaluate the impact of each individual study on the overall effect, sensitivity analysis was applied. We used Begg's test and Egger’s liner regression test to evaluate publication bias^31^. In addition, we performed a trim‐and‐fill approach to obtain an adjusted effect size that takes into account publication bias^32^.

## Results

### Study selection

**Figure 1:**
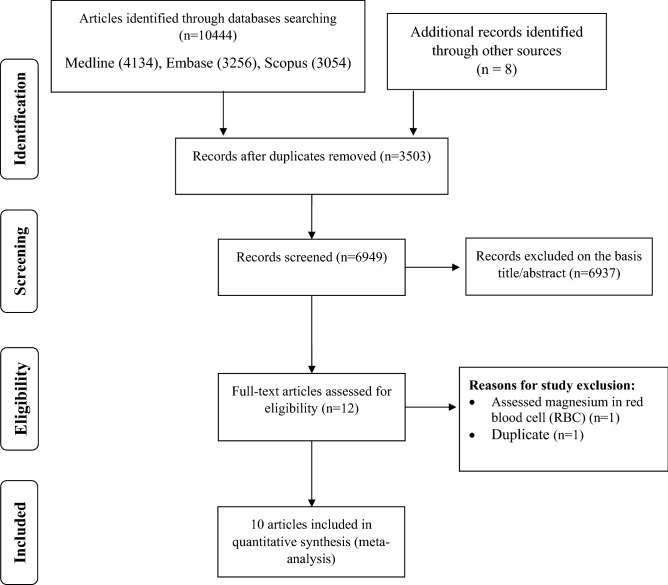
PRISMA flowchart describing the study’s systematic literature search and study selection.

In our primary search 10,452 articles identified from above mentioned databases. After removing 3503 duplicates, 6949 articles retrieved for screening. Based on initial title and abstract screening, we excluded 6933 articles because of the following reasons: unrelated studies (n = 5525), animal studies (n = 1147), review articles (n = 251) and non-English studies (n = 14). By examining the remaining 12 full-text articles, we exclude one study because it assessed the magnesium levels in red blood cell (RBC)^33^. In addition, one similar data was published twice^18,34^ and we included the article that report data in detail and extractable form^18^. Eventually, ten observational studies met our eligibility criteria and include in final analysis (Fig. 1 and Supplementary Table 1)^18–22,35–39^.

### Study characteristics

Characteristics of eligible studies are presented in Table 1. Included studies were published between 1990 and 2019 and the total sample size was 13,455 subjects (2979 prediabetes patients and 10,476 healthy controls). The criteria for diagnosing prediabetes in most studies were criteria recommended by the ADA^35,38,39^ and WHO^19,37^. Of total included studies, one study only enrolled males^20^, while the other studies included both genders. The mean age of all subjects ranged between 29 and 67 years. Out of 10 studies, 3 studies were conducted in China^18,19,36^ and others were conducted in the United states^21^, Netherlands^37^, Sweden^20^, Bangladesh^22^, India^39^, Italy^38^ and Turkey^35^. Two studies assessed magnesium level by inductively coupled plasma spectrometry^18,19^, two studies used atomic absorption^20,36^ and in other studies other methods were used. Six studies used cross-sectional design^20,21,35,37–39^ and the rest of them applied case–control design. The results of Newcastle–Ottawa scale were presented in Table 2. Among ten publications included in the systematic review six studies assigned as a high quality (NOS = 7–9) and four studies assigned as a moderate quality (NOS = 6).

**Table 1:** Characteristics of included studies.

First author (year; location)	Study design	Sample	Population and sample size		Matching	Mean age (years)	Mean BMI	Method of assessment	Magnesium concentration, mmol/L (mean ± SD)	NOS
Zhou (1) (2019; China)	CC	Serum	Prediabetes (IGF)/healthy	Cases: 12	NR	NR	NR	Inductively coupled plasma spectrometer	Cases: 0.88 ± 0.28 Controls: 1.45 ± 0.27	7
				Controls: 50						
Zhou (2) (2019; China)	CC	Serum	Prediabetes (IGT)/healthy	Cases: 15	NR	NR	NR	Inductively coupled plasma spectrometer	Cases: 0.94 ± 0.21 Controls: 1.45 ± 0.27	7
				Controls: 50						
Rahim (2018; Bangladesh)	CC	Serum	Prediabetes/healthy	Cases: 50	Age, Sex	Case: 43.68 Control: 43.26	Case: 27.70 Control: 25.33	NR	Cases: 0.70 ± 0.14 Controls: 0.85 ± 0.15	9
				Controls: 50						
Chen (2017; China)	CC	Plasma	Prediabetes/healthy	Prediabetes: 867	Age, Sex	Case: 52.96 Control: 52.21	Case: 25.09 Control: 23.30	Inductively coupled plasma mass spectrometry	Cases: 0.88 ± 0.12 Controls: 0.91 ± 0.11	8
				Healthy: 2105						
Fang (2016; Chin)	NC	Serum	Prediabetes/healthy	Prediabetes: 145	Gender, Age	Case: 60.23 Control: 60.19	Case: 24.05 Control: 23.23	Flame atomic absorption spectroscopy	Prediabetes: 0.90 ± 3.13 Healthy: 0.97 ± 3.73	9
				Healthy: 145						
Spiga (2019; Italy)	CS	Serum	Prediabetes/healthy	Prediabetes: 224	NR	Prediabetes: 51 Healthy: 44	Prediabetes: 31.5 Healthy: 30.1	Colorimetric method assay	Prediabetes: 0.81 ± 0.07 Healthy: 0.82 ± 0.06	5
				Healthy: 365						
Aksit (2019; Turkey)	CS	Serum	Prediabetes/healthy	Prediabetes: 85	NR	Prediabetes: 34.5 Healthy: 29.75	NR	Photometric method	Prediabetes: 0.84 ± 0.03 Healthy: 0.86 ± 0.05	5
				Healthy: 137						
Kieboom (2017; Netherlands)	CS	Serum	Prediabetes/healthy	Prediabetes: 1346	NR	Prediabetes: 66.6 Healthy: 64.3	Prediabetes: 28.5 Healthy: 26.7	Colorimetric endpoint method and the Roche/Hitachi Cobas c501 Analyzer	Prediabetes: 0.84 ± 0.06 Healthy: 0.85 ± 0.06	6
				Healthy: 7209						
Yadav (2017; india)	CS	Serum	Prediabetes/healthy	Prediabetes: 35	Age	Prediabetes: 36.8 Healthy: 34.8	Prediabetes: 25.35 Healthy: 22.52	Semi-automated analyser	Prediabetes: 0.56 ± 0.15 Healthy: 0.87 ± 0.09	7
				Healthy: 35						
Chambers (1) (2006; USA)	CS	Serum	Prediabetes (African American)/ healthy	Prediabetes: 78	NR	NR	NR	NR	Prediabetes: 0.85 ± 0.08 Healthy: 0.85 ± 0.08	5
				Healthy: 109						
Chambers (2) (2006; USA)	CS	Serum	Prediabetes (Hispanic)/healthy	Prediabetes: 70	NR	NR	NR	NR	Prediabetes: 0.83 ± 0.08 Healthy: 0.83 ± 0.09	5
				Healthy: 169						
Lind (1990; Sweden)	CS	Serum	Prediabetes/healthy	Prediabetes: 52	Age, Sex	NR	NR	Atomic absorption	Prediabetes: 0.79 ± 0.06 Healthy: 0.85 ± 0.06	6
				Healthy: 52						

*BMI* Body Mass Index, *CC* Case–Control, *CS* cross sectional, *NC* Nested case–control, *Mg* Magnesium, *ADA* American Diabetes Association, *WHO* World Health Organization, *NR* Not reported.

**Table 2:** Quality of included studies according to Newcastle–Ottawa Scale (NOS).

Case–control studies									
Publication**s**	Case definition adequate	Representativeness of the cases	Selection of controls	Definition of controls	Comparability of cases and controls	Ascertainment of exposure	Same method of ascertainment	Non-response rate	NOS
Chen et al. (2017)	*	*	–	*	**	*	*	*	8
Fang et al. (2016)	*	*	*	*	**	*	*	*	9
Rahim et al. (2018)	*	*	*	*	**	*	*	*	9
Zhou et al. (2019)	*	*	*	*	_	*	*	*	7

Cross sectional studies									
Publications	Representativeness of the sample	Sample size	Non-respondents	Ascertainment of the exposure	Comparability	Ascertainment of outcome	Statistical test		NOS
Kieboom et al. (2017)	*	–	*	*	–	**	*		6
Lind et al. (1990)	*	–	*	*	**	–	*		6
Yadav et al. (2017)	*	–	*	*	*	**	*		7
Spiga et al. (2019)	*	–	*	*	–	*	*		5
Chambers et al. (2006)	*	–	*	*	–	*	*		5
Aksit et al. (2019)	*	–	*	*	–	**	*		5

### Overall meta-analysis

**Figure 2:**
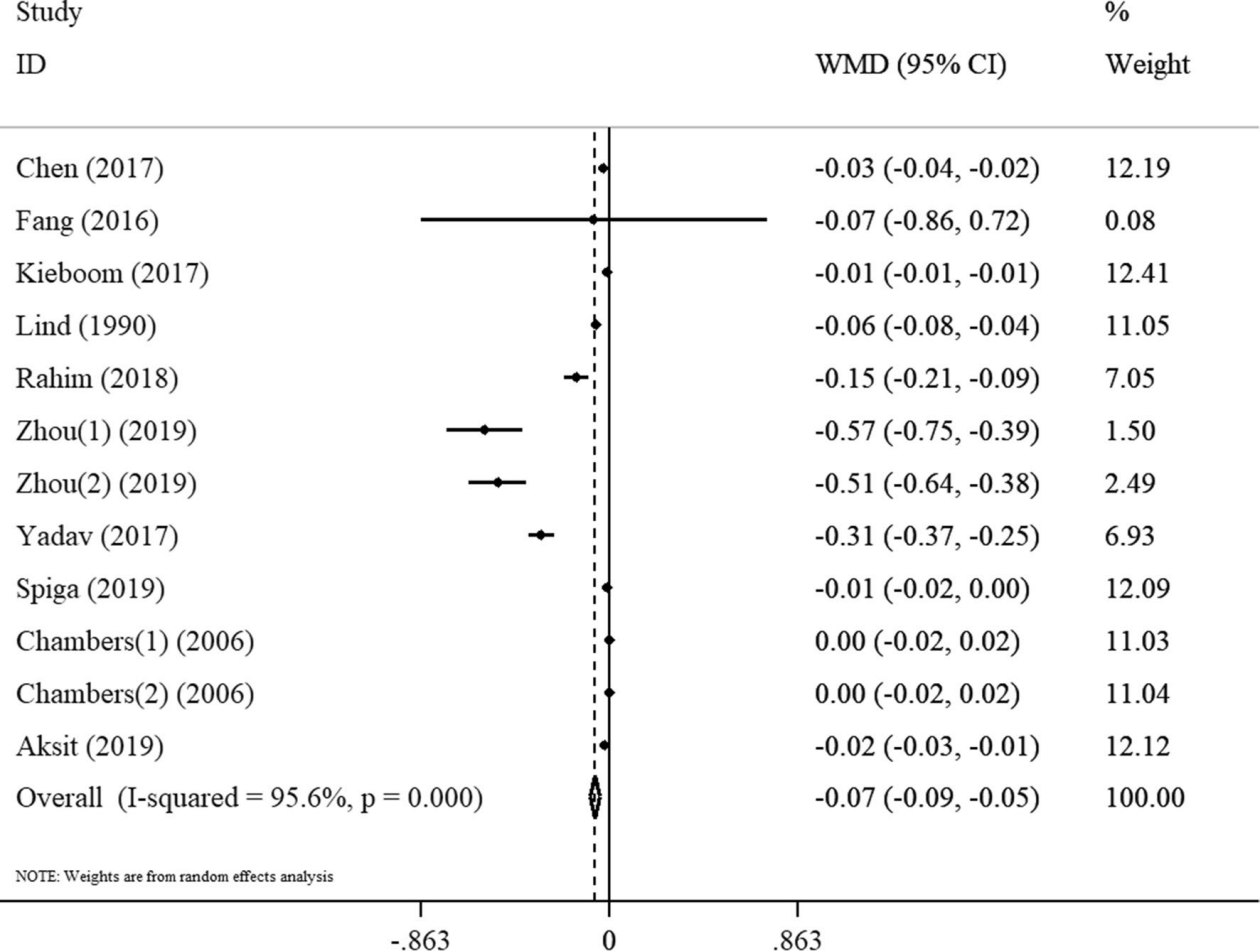
Forest plot for the association between magnesium level and prediabetes expressed as mean difference between case and control groups. The area of each square is proportional to the inverse of the variance of the WMD. Horizontal lines represent 95%Cis. Diamonds represent pooled estimates from random-effects analysis. WMD, weighted mean difference.

Forest plot showing the association between magnesium level and prediabetes depicted in Fig. 2. The random effects meta-analysis indicated that subjects with prediabetes had a significantly lower serum magnesium concentration compared with their healthy controls (WMD =  − 0.07 mmol/L; 95% CI: − 0.09, − 0.05 mmol/L, P < 0.001, Fig. 2). However, evaluation of studies in terms of heterogeneity demonstrated a high degree of heterogeneity (I^2^ = 95.6%, P < 0.001). Following the subgroup analysis based on sample type (serum and plasma), publication year (≤ 2010 and > 2010) and study design (case–control and cross-sectional), we could not find the origin of heterogeneity (Table 3). The subgroup analysis showed that magnesium concentrations in prediabetic patients were significantly lower than those in healthy individuals in studies published before 2010 (WMD = − 0.12 mmol/L, 95% CI − 0.14, − 0.09). However, there was no significant difference in magnesium concentration when compared between healthy controls and prediabetes subjects in studies published 2010 onwards (WMD = − 0.02 mmol/L, 95% CI − 0.06, 0.02). The results of subgroup analysis based on the quality of the study also indicated that compared to healthy individuals, the concentration of magnesium in prediabetic individuals is lower in high quality studies (WMD =  − 0.87 mmol/L, 95% CI − 1.29, − 0.45) than in moderate quality studies (WMD =  − 0.37 mmol/L, 95% CI − 0.61, − 0.13). Moreover, the analysis showed that magnesium concentrations in the pre-diabetic group were lower than in healthy subjects in European studies (WMD =  − 0.35 mmol/L, 95% CI − 0.62, − 0.07), while in US studies, magnesium concentrations were not significantly different between the healthy and pre-diabetic groups (WMD =  − 0.00 mmol/L, 95% CI − 0.20, 0.20). Our analysis also highlighted that studies were performed in another region reported the lowest magnesium concentration with prediabetes compared to their healthy counterparts (WMD =  − 1.09 mmol/L, 95% CI − 1.57, − 0.61). Subgroup analysis of our study based on comparability also showed that magnesium concentrations in prediabetics in studies that have no or incomplete adjustment (WMD =  − 0.74 mmol/L, 95% CI − 1.08, − 0.40) were lower than studies had complete adjustment (WMD =  − 0.53 mmol/L, 95% CI − 0.91, − 0.15). Sensitivity analysis showed that the omission of each individual study had no effect on the overall results.

**Table 3:** Subgroup analysis to assess the magnesium concentrations in subjects with prediabetes.

Sub grouped by	No	WMD (95% CI)	P-value	P-Heterogeneity	I^2^ (%)	P- between subgroup heterogeneity
**Publication year**						
≤ 2010	2	− 0.02 (− 0.06, 0.02)	0.318	< 0.001	88.4%	0.362
> 2010	8	− 0.12 (− 0.14, -0.09)	< 0.001	< 0.001	97.8%	
**Sample**						
Serum	9	− 0.10 (− 0.12, − 0.07)	< 0.001	< 0.001	97.3%	< 0.001
Plasma	1	− 0.03 (− 0.04, -0.02)	< 0.001	–	0%	
**Study design**						
Case–control	3	− 0.25 (− 0.45, -0.05)	0.016	< 0.001	98.6%	< 0.001
Cross- sectional	6	− 0.09 (− 0.11, − 0.06)	< 0.001	< 0.001	95.1%	
Nested case–control	1	− 0.07 (− 0.86, 0.72)	0.863	–	0%	
**Region**						
US	2	0.00 (− 0.20, 0.20)	1.00	1.00	0%	< 0.001
European	2	− 0.35 (− 0.62, 0.07)	0.015	< 0.001	87.3%	
Others	6	− 1.09 (− 1.57, − 0.61)	< 0.001	< 0.001	94.7%	
**Quality score**						
High	7	− 0.87 (− 1.29, − 0.45)	< 0.001	< 0.001	94.2%	0.02
Moderate	3	− 0.37 (− .0.61, − 0.13)	0.002	< 0.001	84.7%	
**Comparability**						
Complete adjustment	4	− 0.53 (− 0.91, − 0.15)	0.006	< 0.001	89.8%	0.05
No or incomplete adjustment	6	− 0.74 (− 1.08, − 0.40)	< 0.001	< 0.001	93.8%	

*WMD* weighted mean difference.

### Publication bias

**Figure 3:**
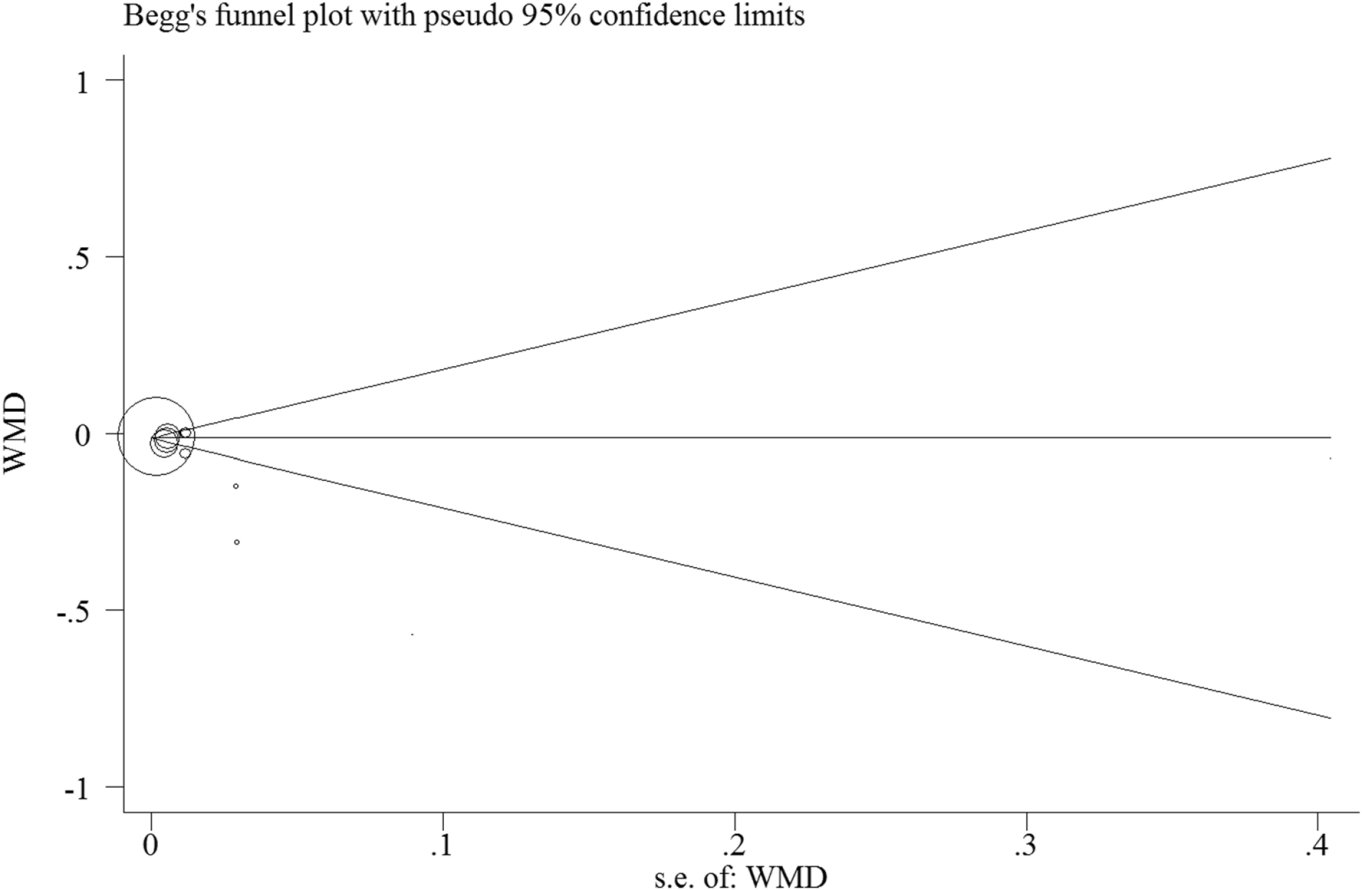
Funnel plot of the weighted mean difference (WMD) versus the s.e. of the weighted mean difference (WMD). All statistical analyses were performed using Stata version 14 (StataCorp. 2015. Stata Statistical Software: Release 14. College Station, TX: StataCorp LP, www.stata.com).

Although a comprehensive search was performed to reduce the possibility of publication bias, we also use Begg’s and Egger’s test to detect any potential publication bias. Begg’s test did not find significant publication bias (P = 0.1) (Fig. 3), but Egger's test indicated significant publication bias (P = 0.008). Finally, trim-and-fill analysis indicated no trimming and data unchanged.

## Discussion

The role of magnesium in the management of chronic diseases such as metabolic syndrome, cardiovascular disease, cerebrovascular accident, hypertension and type 2 diabetes mellitus has received a great attention in recent decades^40–45^. Hypomagnesemia is one of the causes of insulin resistance, high blood glucose, and cardiovascular complications of diabetes^46,47^. Patients with diabetes had increased magnesium excretion in the urine, which is due to hyperglycemia, hyperfiltration, and the effect of insulin on the renal channels of magnesium^48^. Although among people with prediabetes serum glucose levels are below the threshold for magnesium extraction in the urine and they are unlikely to affect serum magnesium levels^37^, but a number of studies have shown that magnesium levels in subjects with prediabetes are also lower than healthy individuals^19,22,34^. Previous meta-analysis has reported an inverse relationship between magnesium levels and magnesium intake with the risk of diabetes, and also confirmed the beneficial effect of magnesium supplementation in the management of glucose disorders^8,9,16,49^.

Although many studies have examined the association between magnesium and diabetes, there are limited studies on the association of magnesium with prediabetes and its progression to diabetes. To the best of our knowledge, this is the first systematic review and meta-analysis to examine the association between serum magnesium levels and prediabetes. The present meta-analysis of ten observational studies involving 2979 cases and 10,476 controls detected that serum magnesium levels are lower in prediabetes patients compared with their healthy controls. Significant heterogeneity was observed among the included studies. Following the subgroup analysis based on sample type, publication year and study design, we could not identify the source of heterogeneity, which may be attributed to other study parameters such as the magnesium assessment methods, criteria for diagnosing prediabetes, and ethnic populations. Due to lack of sufficient information about them, we could not perform subgroup analysis and find the source of heterogeneity.

Magnesium plays an important role in the activity of more than 300 enzymes, including all enzymes that use or synthesize adenosine triphosphate (ATP), as well as enzymes effective in glucose metabolism^50,51^. Various studies have confirmed the fundamental role of magnesium in glucose metabolism, including cellular uptake of glucose, glucose excretion, insulin secretion and insulin function^52–55^. Among patients with impaired glucose metabolism, there is a higher renal magnesium wasting due to reduced tubular magnesium reabsorption resulting from glucose-induced osmotic diuresis^20,48,56^. Low levels of magnesium can inhibit phosphorus-dependent reactions and the activity of many enzymes involved in glucose metabolism, thereby reducing insulin secretion, preventing cellular glucose uptake and promoting the development of metabolic glucose disorders^50,57^. Magnesium deficiency can cause serious complications in glucose disorders, such as cardiac arrhythmia, hypertension and myocardial infarction, thus, considering magnesium status can be important in the management of prediabetes and its progression to diabetes^58,59^.

Several limitations of our meta-analysis should be acknowledged. Firstly, the design of all studies included in the current meta-analysis are observational, so we could not infer a causal association between serum magnesium level and prediabetes. Secondly, most of included studies did not make adjustment for the potential confounders, especially age and gender. Thus, the residual confounder may affect the results. Thirdly, the diagnostic criteria for prediabetes were different in the studies that could affect the outcome. Fourthly, we use the "mean difference" of serum magnesium levels as an effect size which are not the best effect measure compared to odds ratio or hazard ratio with 95% CI to evaluate the relationship between outcome and exposure. Fifthly, the included studies did not measure the magnesium intake which could be attributed to the serum magnesium levels. In addition, we have excluded studies published in a non-English language that may affect the final outcome. Finally, we observed a significant heterogeneity among included studies. However, our attempts to detect the potential source of heterogeneity through different subgroup analysis were unsuccessful. Therefore, the results should be interpreted with caution.

## Conclusions

The present meta-analysis indicated that circulating magnesium levels in people with prediabetes were significantly lower than healthy controls, confirming that magnesium deficiency may play a role in development of prediabetes. However, it should be noted that due to the small estimate effect, the results should be interpreted with caution. Assessment of magnesium levels in subjects with prediabetes and improvement of possible deficiencies may prevent its progression to diabetes. Further prospective cohort studies with larger sample size and robust design are warranted to confirm present findings.

## Supplementary Information

Supplementary Tables.

Supplementary Information.
