# Supplementary material for: Association between magnesium concentrations and prediabetes: a systematic review and meta-analysis
Source: Sci Rep. 2021 Dec 22;11:24388. doi: 10.1038/s41598-021-03915-3 (PMC8695575; doi:10.1038/s41598-021-03915-3)
Supplement: Supplementary file 1 — Supplementary Tables. [file 41598_2021_3915_MOESM1_ESM.docx]

| **Database** | **key terms and the queries** | Number of studies |
| --- | --- | --- |
| PubMed | #1 magnesium[MeSH Terms] OR magnesium[Title/Abstract]  #2 ("Insulin Resistance"[Title/Abstract])) OR ("Glucose Metabolism Disorders"[Title/Abstract])) OR ("prediabetic state"[Title/Abstract])) OR ("impaired glucose tolerance"[Title/Abstract])) OR ("prediabetes"[Title/Abstract])) OR ("pre-diabetes"[Title/Abstract])) OR ("prediabetic state"[Title/Abstract])) OR ("hyperglycaemia"[Title/Abstract])) OR ("borderline diabetes"[Title/Abstract])) OR ((((((Diabetes[Title/Abstract]) ) OR (hyperglycemia[Title/Abstract])) OR (Glycemia[Title/Abstract])) OR (Glycemic[Title/Abstract]))) OR (((("Diabetes Mellitus"[Mesh] OR "Insulin Resistance"[Mesh]) OR "Glucose Metabolism Disorders"[Mesh]) OR "Prediabetic State"[Mesh]))  #3 #1 AND #2 | 4134 |
| Scopus | ( TITLE-ABS-KEY ( " magnesium" ) AND ( TITLE-ABS-KEY ( "Diabetes Mellitus" OR ("Insulin Resistance" OR "Glucose Metabolism Disorders" OR "prediabetic state" OR "impaired glucose tolerance" OR "prediabetes" OR "pre-diabetes" OR "hyperglycaemia" OR "borderline diabetes" OR " Diabetes " OR " hyperglycemia "OR " Glycemia" OR " Glycemic " )) ) | 3054 |
| Embase | #1 'magnesium':ti,ab,kw OR 'magnesium'/exp  #2 'Diabetes Mellitus' :ti,ab,kw OR ' Insulin Resistance ':ti,ab,kw OR ' Glucose Metabolism Disorders'/exp OR ' prediabetic state ':ti,ab,kw OR ' impaired glucose tolerance ':ti,ab,kw OR 'thyroid'/exp OR ' pre-diabetes':ti,ab,kw OR ' hyperglycaemia ' OR ' borderline diabetes ':ti,ab,kw OR ' Diabetes :ti,ab,kw OR ' hyperglycemia ':ti,ab,kw OR 'Glycemia':ti,ab,kw OR ' Glycemic ':ti,ab,kw  #3 #1 AND #2 | 3256 |

**Supplementary Table 1.** Search strategies including the key terms and the queries for each database

| **Supplemental Table 2**: Reason for exclusion of retrieved articles | |
| --- | --- |
| References | Reason for exclusion |
| 1. Magnesium status and dietary intake of mid-old people in a rural area of China | Assessed magnesium in red blood cell (RBC) |
| 1. Associations of serum and urinary magnesium with the pre-diabetes, diabetes and diabetic complications in the Chinese Northeast population | No relevant exposure reported |
